# Heart Under Pressure: From Scene to Scalpel in Cardiac Tamponade From a Left Ventricular Stab Injury

**DOI:** 10.7759/cureus.116967

**Published:** 2026-09-26

**Authors:** Miguel Gonçalves Pereira, Adrian Bolnberger, Christos Farazi-Chongouki, Maria E Batista, Chido Nyatsambo, Riaan Pretorius

**Affiliations:** 1 Critical Care Medicine Department, Unidade Local de Saúde da Guarda EPE, Guarda, PRT; 2 Anaesthesia, Intensive Care Medicine and Pain Medicine Department, Vienna General Hospital - Medical University of Vienna, Austrian South African Medical Exchange (ASAMEx), Vienna, AUT; 3 General Surgery Department, Thriasio General Hospital, Athens, GRC; 4 Critical Care Medicine Department, Hospital Curry Cabral, Unidade Local de Saúde São José, Lisbon, PRT; 5 Trauma Unit, Chris Hani Baragwanath Academic Hospital, Johannesburg, ZAF

**Keywords:** cardiac tamponade, penetrating cardiac injury, penetrating chest trauma, pericardial effusion, sternotomy, ventricle injury

## Abstract

Penetrating cardiac injuries carry exceptionally high mortality, especially when the left ventricle is involved, and survival hinges on rapid diagnosis and immediate surgical intervention. We describe a case of penetrating cardiac trauma with acute tamponade, emphasizing the role of coordinated care from scene to operating theatre. A 30-year-old male presented with multiple precordial stab wounds, in hemorrhagic shock with severe hypoxemia; prehospital teams prioritized rapid stabilization and transport. On arrival, extended focused assessment with sonography for trauma (eFAST) identified left hemopneumothorax and circumferential pericardial effusion consistent with tamponade, and the massive transfusion protocol was activated. Emergency median sternotomy revealed a grade IV left ventricular laceration, which was repaired primarily without cardiopulmonary bypass, and the patient recovered well, with progressive hemodynamic stability and favorable metabolic parameters postoperatively. This case illustrates that survival from penetrating left ventricular injury requires rapid diagnosis via point-of-care ultrasound, immediate median sternotomy, and intensive postoperative management, and demonstrates that favorable outcomes are achievable through coordinated multidisciplinary care within an organized trauma system. Penetrating cardiac injuries remain time-critical emergencies, and early recognition via point-of-care ultrasound, prompt surgical access, and a well-rehearsed trauma protocol can achieve survival even when severe ventricular injuries are present.

## Introduction

Penetrating chest trauma, although less frequent than blunt thoracic injury, is associated with a higher risk of life-threatening complications, particularly when cardiac structures are involved. Penetrating cardiac injuries account for approximately 6% of penetrating thoracic trauma cases and carry significant mortality, with prehospital mortality approaching 90% in some series - largely because resuscitative efforts in the field are of limited benefit [1,2]. Death typically results from cardiac tamponade, massive hemorrhage, or rapid circulatory collapse [3].

Left ventricular involvement confers a particularly poor prognosis. Survival rates for isolated left ventricular injuries range from 26% to 28%, compared with approximately 31-79% for right ventricular wounds, reflecting the greater hemodynamic consequence of left-sided injuries [4]. Survival following penetrating cardiac injury is closely linked to a coordinated chain of care that includes early recognition at the scene, rapid transport to a trauma centre, prompt diagnosis, and immediate surgical intervention. Prehospital decision-making and effective communication with the receiving hospital play a pivotal role in optimizing outcomes [1,5].

It is worth situating this case within the broader history of cardiac surgery, which itself originated from the operative management of penetrating cardiac trauma. In an era with no cardiopulmonary bypass, no antibiotics, and no dedicated cardiac anaesthesia, Ludwig Rehn performed the first successful cardiac suture on 9 September 1896 in Frankfurt, repairing a stab wound to the right ventricle of a 22-year-old gardener injured two days earlier; the patient survived, and by 1907 Rehn had accumulated 124 cardiorrhaphies with a reported 60% survival rate, in stark contrast to the near-uniformly fatal course of unoperated penetrating cardiac wounds at the time [6,7]. This single operation is widely regarded as the founding event of modern cardiac surgery, and the principle it established - that even a severe, delayed-presenting penetrating cardiac wound can be survived once the pericardium is opened and the heart repaired promptly - remains directly relevant to the present report. Subsequent technical milestones in this field, including Duval's description of the median sternotomy [8] and Beck's characterisation of the pericardial tamponade triad and of coronary-sparing suture technique [9], were likewise derived from the surgical management of civilian stabbing injuries, decades before elective cardiac surgery became feasible [10].

In this report, we present a case of penetrating left ventricular injury with acute tamponade. The patient survived through coordinated prehospital and in-hospital surgical management. This case demonstrates how protocol-driven care and timely intervention can result in a favorable outcome, even in severe cardiac trauma.

Cardiac tamponade is itself the proximate cause of death in many of these cases: as blood accumulates within the fixed, non-compliant pericardial sac, rising intrapericardial pressure progressively impairs diastolic filling of the cardiac chambers, reducing stroke volume and cardiac output despite compensatory tachycardia - a form of obstructive shock that, if unrecognised, rapidly progresses to pulseless electrical activity and death. Against this background, and against the 26-28% survival reported for isolated left ventricular injuries [4], the present case is notable for combining a severe (American Association for the Surgery of Trauma (AAST) Grade IV), transmural left ventricular laceration with profound hemorrhagic and obstructive shock, a fully reconstructed prehospital-to-operative timeline, and a favourable outcome achieved without cardiopulmonary bypass. Few reports of penetrating left ventricular trauma document this combination of injury severity, physiological derangement, and granular time-interval data alongside survival; we believe this level of detail is of particular educational value to prehospital and trauma teams managing similar presentations, illustrating both the physiological tolerance for a finite period of untreated tamponade and the specific chain-of-care elements that made survival possible in this instance.

This case report has been reported in line with the Surgical CAse REport (SCARE) checklist 2025 guidelines.

## Case presentation

Prehospital management

A critical response vehicle was dispatched at 11:53 to a stabbing in Soweto, Johannesburg, arriving on scene at 12:23. A Gauteng Provincial Government ambulance had reached the patient 10 minutes earlier and started basic care: wound dressing, IV access, and 500 mL crystalloid.

The patient was a 30-year-old male with no prior medical history. On assessment, he was drowsy with a Glasgow Coma Scale of 12, hypotensive (71/53 mmHg), tachycardic (93 bpm), and hypoxemic (SpO₂ 91% on room air). Three stab wounds were identified: one to the anterior chest wall 2 cm medial to the left nipple (left precordium, fourth intercostal space) (Figure 1), one to the left forearm, and one at the inferior border of the left scapula. Clinical examination revealed diminished breath sounds on the left, audible bubbling from the chest wound, muffled heart sounds, and poor peripheral perfusion.

**Figure 1:**
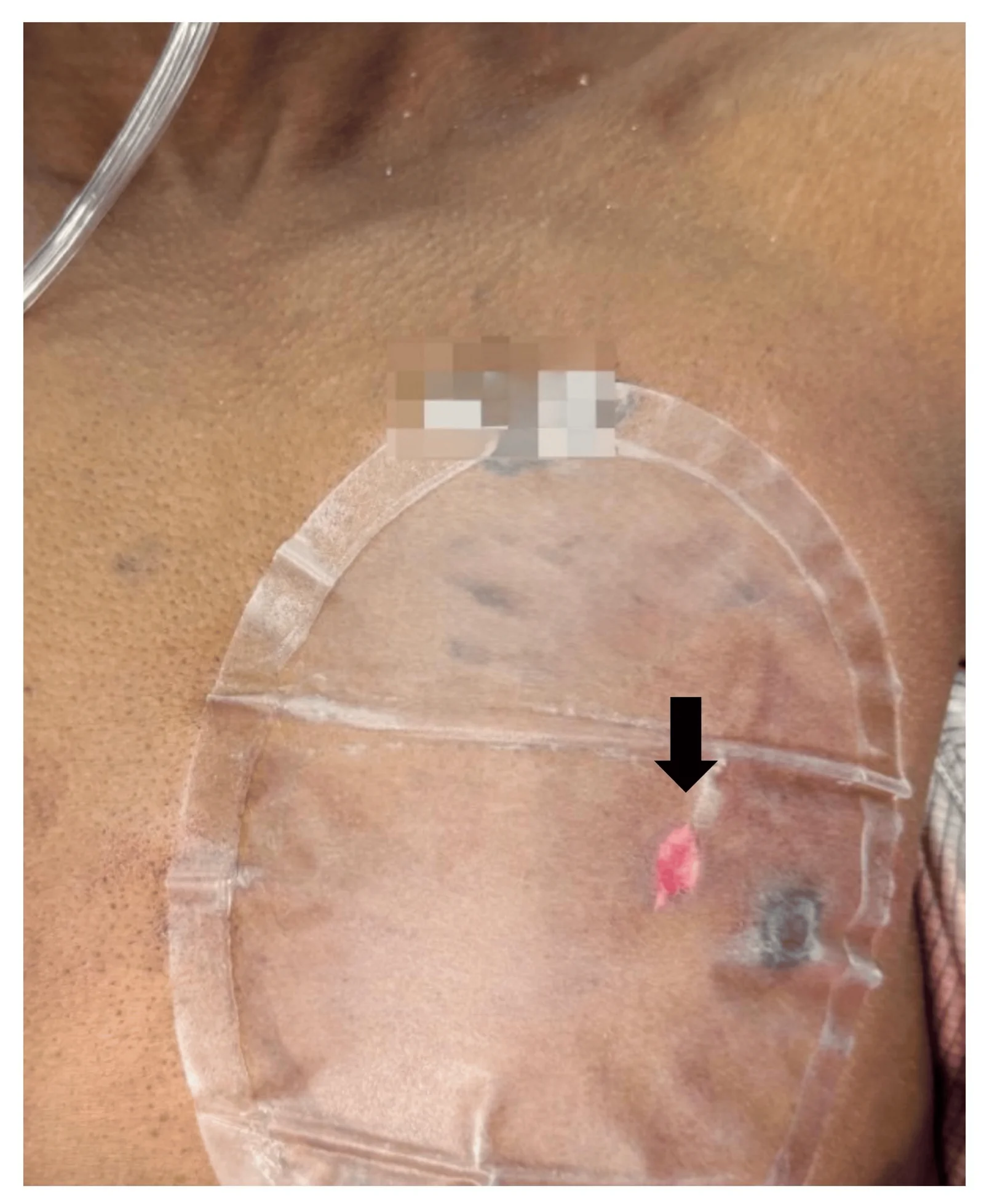
Anterior left chest stab wound under occlusive chest seal

Non-vented occlusive chest seals were applied. A second IV line was placed, and an additional 500 mL crystalloid was given (1 L total). Oxygen was started at 15 L/min. Morphine 5 mg and metoclopramide 10 mg were administered IV. Rapid transport to a level I trauma center - Chris Hani Baragwanath Academic Hospital - was prioritized while hemodynamic instability persisted.

In-hospital management

The patient arrived at the Trauma Resuscitation Unit at 12:47. His airway was patent, and SpO₂ had improved to 96% on high-flow oxygen, but respiratory rate had dropped to 10 breaths/min. Blood pressure remained low (100/37 mmHg) with a heart rate of 110-115 bpm. Extended focused assessment with sonography for trauma (eFAST) showed left hemopneumothorax and a 1.8 cm circumferential pericardial effusion consistent with tamponade. Still or video images from this examination were not archived, reflecting the priority given to immediate resuscitation and damage-control decision-making in a critical, rapidly evolving clinical situation. Venous blood gas revealed severe acidosis: pH of 7.05, pCO₂ of 68 mmHg, lactate of 7.8 mmol/L, BE of -12.8, and HCO₃ of 18.8 mmol/L. GCS decreased to 10/15 (E3 V2 M5).

One unit of O-negative packed red cells and two freeze-dried plasma packs were administered immediately; platelets were unavailable at the time of initial resuscitation. Massive transfusion protocol was activated, with further red cell and plasma units administered intraoperatively according to institutional massive-transfusion ratios (exact total intraoperative volumes were not separately tabulated in the record; see Limitations), and tranexamic acid 1 g was administered. Mild hypocalcemia was corrected with 10% calcium chloride. A 36Fr chest drain was inserted in the left fourth intercostal space, a central line was placed, and adrenaline infusion started, reaching 0.09 µg/kg/min. The operating theatre was alerted for emergency sternotomy.

In theatre, the patient remained unstable (BP of 83/50 mmHg, HR of 127 bpm), with a pre-intubation GCS score of 10. Sternotomy began at 13:36 (Figure 2), following unavoidable delays related to operating theatre availability and activation logistics. The exact time of the assault itself was not witnessed and could not be established with certainty; using the emergency call (11:53) as the earliest verifiable reference point, the interval from alarm to skin incision was approximately 1 hour and 43 minutes. Taking the emergency call (11:53) as the reference point, the key intervals were: alarm to EMS (CR1) on scene, 30 minutes; alarm to arrival at the Trauma Resuscitation Unit, 54 minutes; and alarm to skin incision (sternotomy), approximately 1 hour and 43 minutes. A discrete ‘operating room entry’ time distinct from skin incision was not separately recorded. A 2 cm stellate pericardial laceration over the left ventricle was found-Grade IV by the AAST Heart Injury Scale. The laceration was transmural, communicating directly with the left ventricular cavity, which accounted for the rapid accumulation of pericardial blood and the tamponade physiology seen on eFAST; the left anterior descending coronary artery and its diagonal branches were identified running close to, but not through, the laceration, and were visually intact throughout the repair, so that no coronary ligation or coronary-sparing mattress sutures were required. The pericardium was evacuated, and manual cardiac massage was performed. The left ventricular injury was repaired with 2-0 Prolene sutures (Figures 3-4). Repair was performed with the heart beating, without institution of cardiopulmonary bypass, consistent with standard practice for isolated ventricular lacerations that do not involve the proximal coronary arteries or cardiac valves. Left hemothorax was drained (approximately 2 L), and blood was autotransfused using cell salvage. Three drains were placed: one pericardial and two pleural (apical and basal) (Figure 5). Direct inspection of the left lung through the sternotomy and thoracostomy tract showed no parenchymal laceration; the associated hemopneumothorax was therefore attributed to the open chest wound and to blood tracking from the wound tract and pericardial space into the pleural cavity, rather than to a pulmonary injury (see Discussion). After hemostasis, adrenaline was weaned to 0.04 µg/kg/min. Immediate postoperative blood gas showed modest but progressive improvement: pH of 7.16, lactate of 7.0 mmol/L, and HCO₃ of 17.1 mmol/L. The patient was transferred to high-care due to lack of ICU beds.

**Figure 2:**
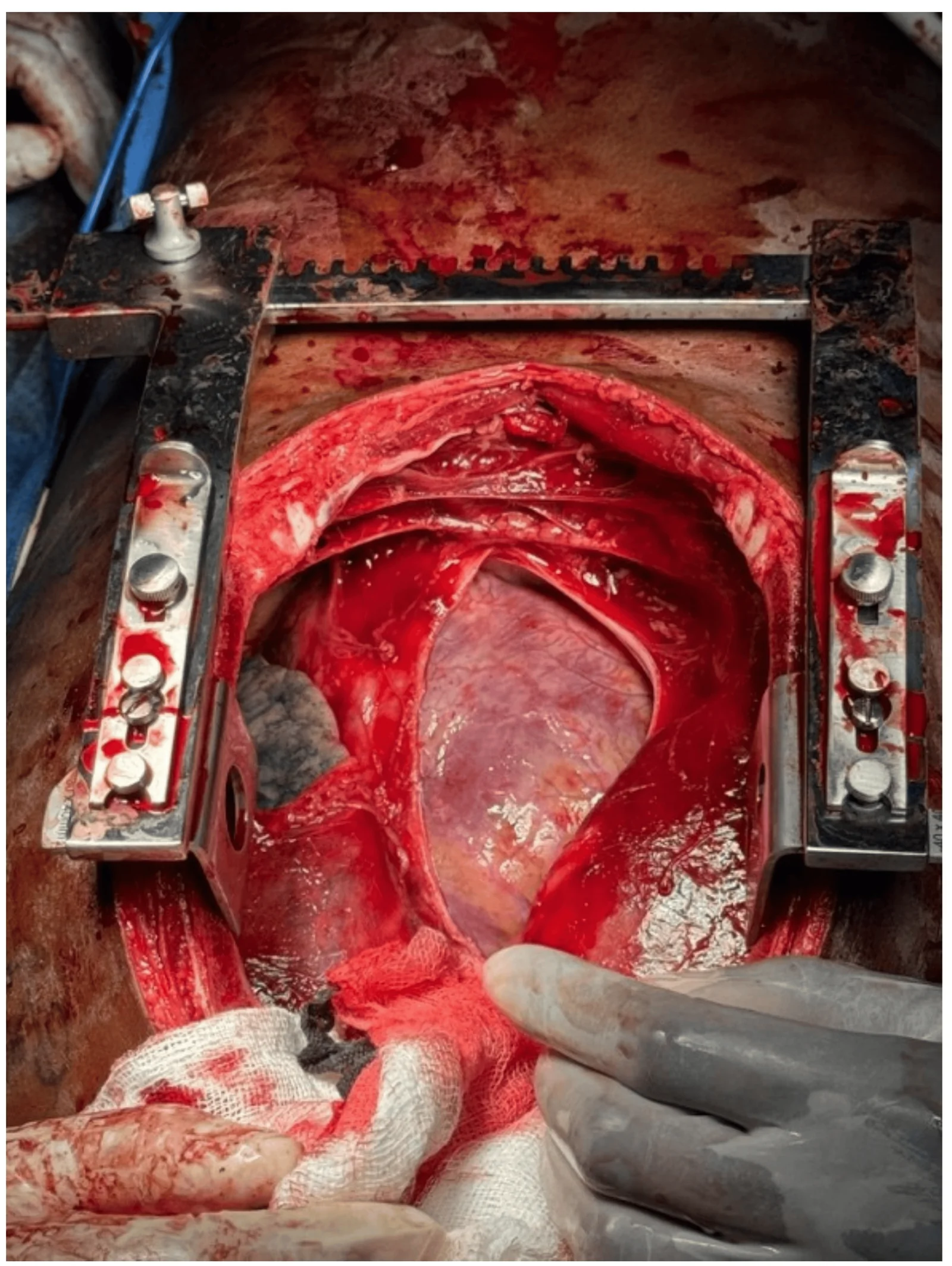
Median sternotomy with pericardial and pleural cavity exposure

**Figure 3:**
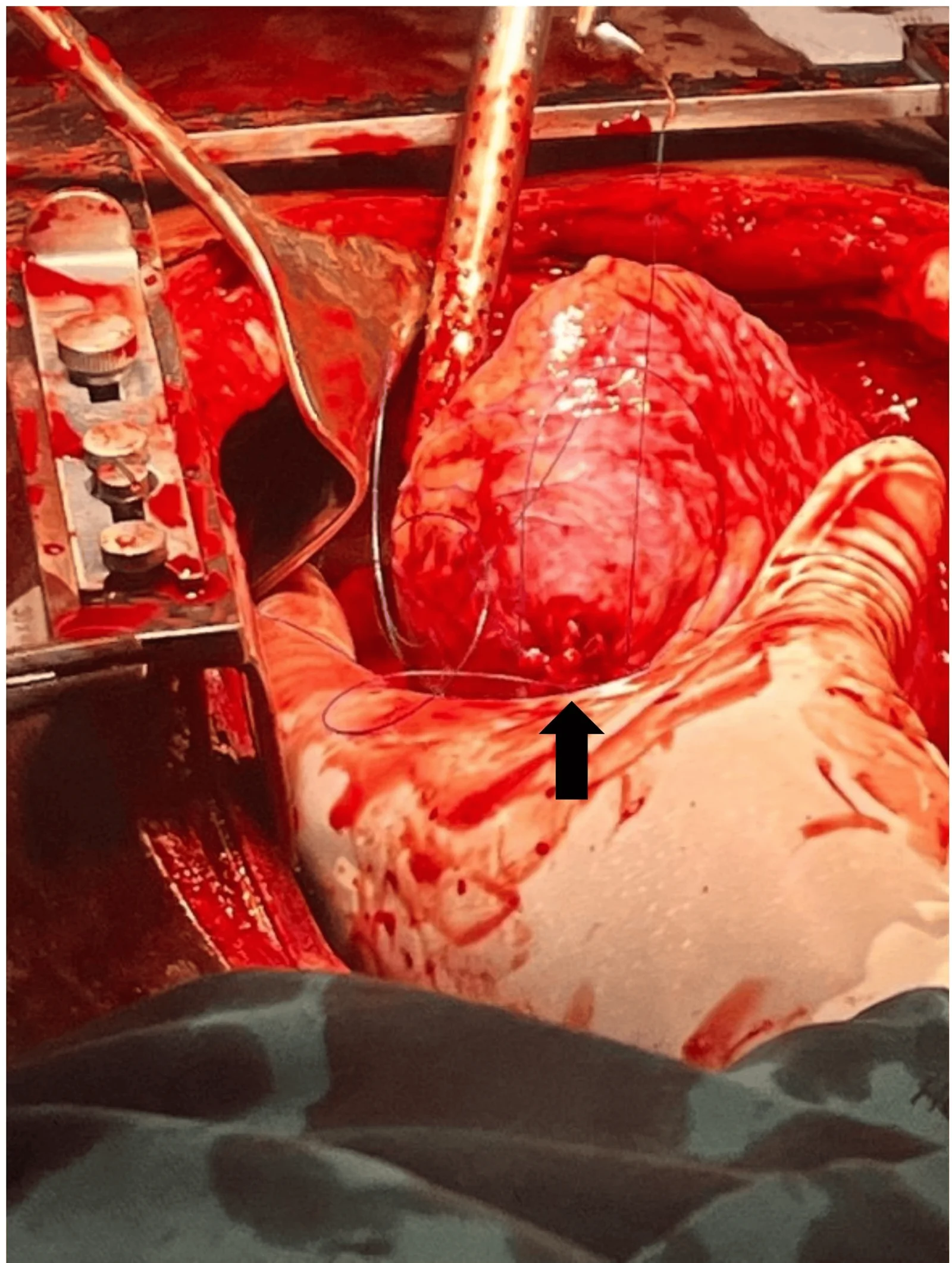
Left ventricle suturing

**Figure 4:**
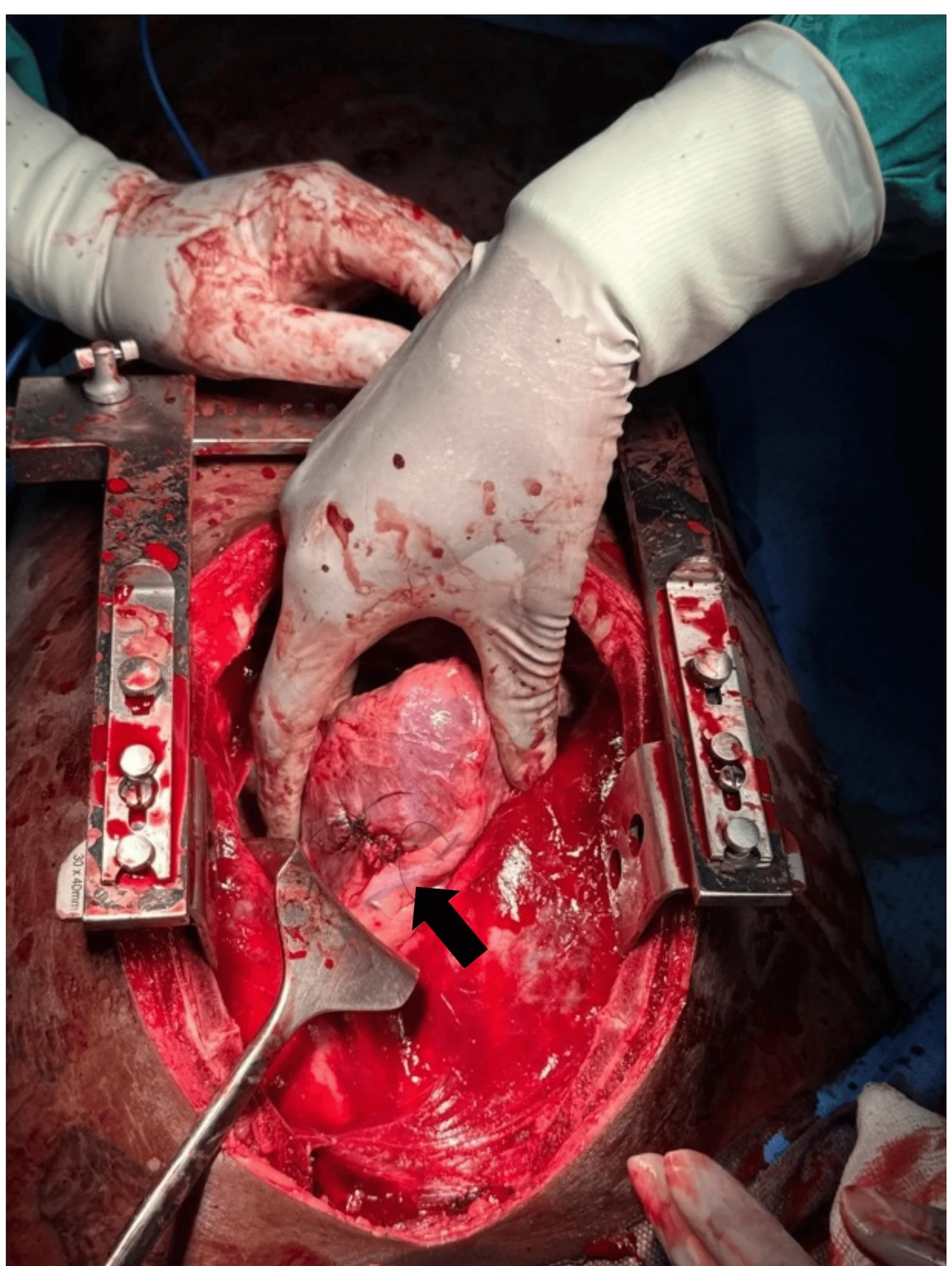
Stellate left ventricular lesion after prolene suturing

**Figure 5:**
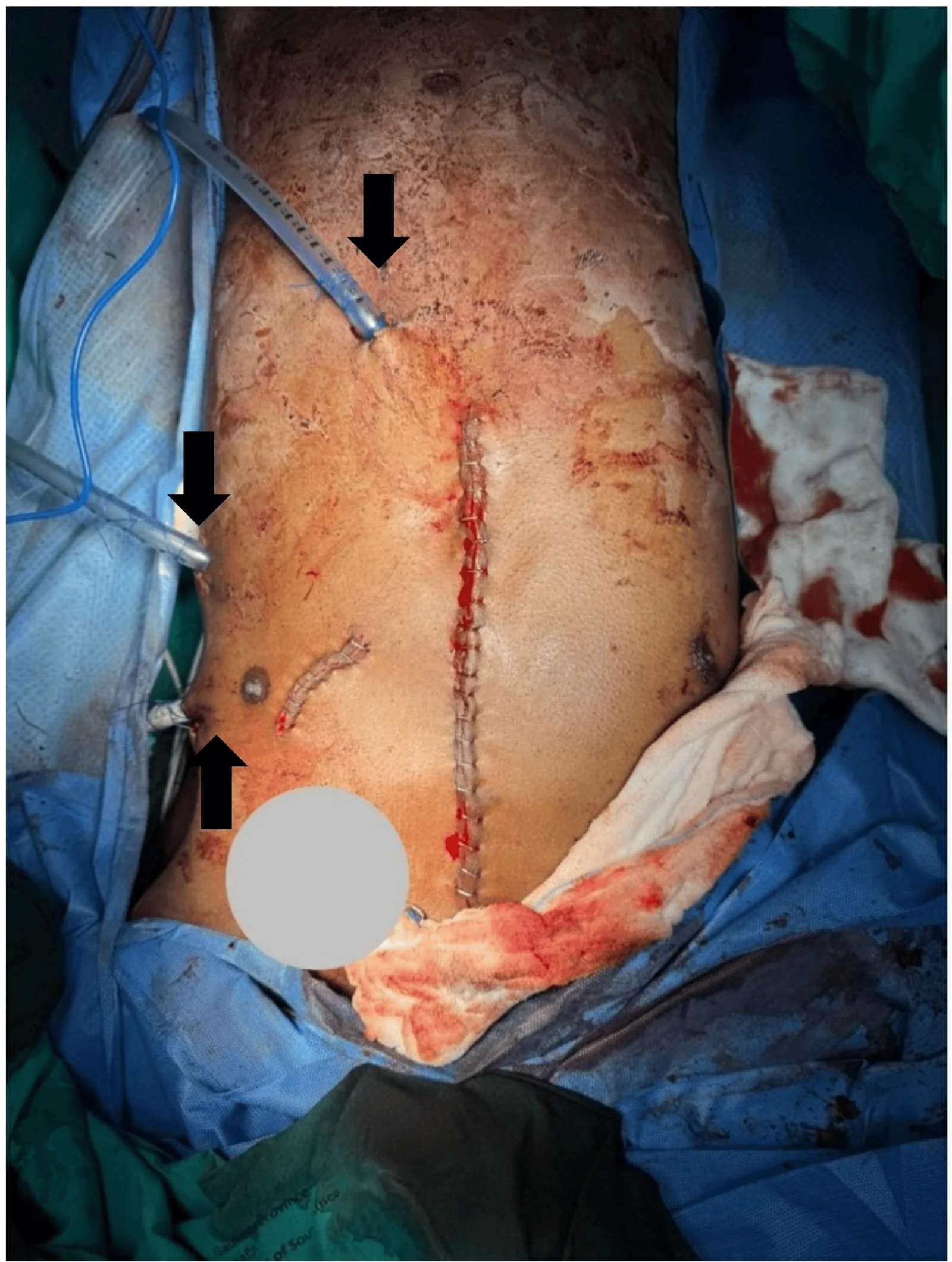
Thoracic drains

The overall timeline of prehospital and in-hospital events, from initial call to discharge, is summarized in Figure 6.

**Figure 6:**
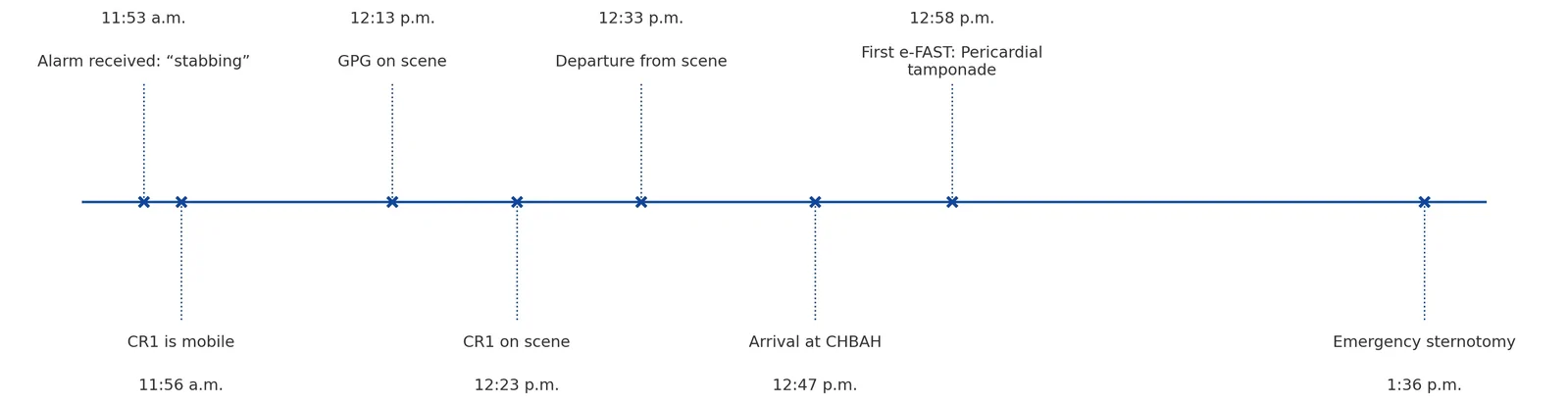
Case timeline summarizing the clinical events and interventions CR1 - Critical Response Vehicle 1 (operated by UREMS and ASAMEx Paramedic Crew); GPG - Gauteng Provincial Ambulance. Times before the emergency call (11:53) - including the exact time of the stab injury itself - were not documented and are not depicted; the alarm-to-incision interval was approximately 1 hour and 43 minutes. Cardiac repair was performed without cardiopulmonary bypass.

On Day 3, echocardiography demonstrated good LV function with minimal pericardial effusion. Drains came out on Days 4 and 9. The patient was discharged home on Day 11. Despite repeated attempts, the patient could not be reached for structured outpatient follow-up thereafter, and no clinical or echocardiographic data beyond Day 11 are available (see Limitations).

## Discussion

Penetrating cardiac injuries carry high mortality even in experienced trauma centers, and left ventricular involvement typically results in rapid hemodynamic collapse through exsanguination or acute tamponade. Survival is closely linked to timely diagnosis and immediate operative intervention [11-14].

Across the wider literature, survival after penetrating cardiac injury is consistently linked to a recognisable set of factors: the presence of pericardial tamponade rather than free pericardial rupture into the pleural or mediastinal space (77% vs 11% survival in one series) [10]; right-sided rather than left-sided chamber injury (up to 79% vs 26-31% survival) [4,10]; single- rather than multi-chamber involvement [5]; signs of life on arrival, such as a palpable pulse, pupillary reactivity, or organised cardiac electrical activity [10]; a stabbing rather than a gunshot mechanism, which allows a substantially longer physiological window before exsanguination [5]; prehospital transport time under approximately nine minutes to a trauma centre [10]; a systolic blood pressure of 60 mmHg or more in the field and on arrival [10]; and, where prehospital cardiopulmonary resuscitation is required, a duration under three minutes in unintubated patients [10]. Our patient met several of these favourable criteria: a stabbing (not gunshot) mechanism, single-chamber involvement with tamponade physiology rather than free rupture, a recordable blood pressure and preserved (if reduced) consciousness throughout transport, and no requirement for prehospital cardiopulmonary resuscitation.

Several factors contributed to the favorable outcome in this case. Despite initial stabilization at the scene, the patient deteriorated rapidly - a pattern characteristic of cardiac injury, where clinical stability is often transient and deceptive. Point-of-care ultrasound identified both hemopneumothorax and pericardial effusion, directing the patient immediately to surgery [15,16]. Despite the absence of prehospital notification, the trauma team responded promptly upon the patient's arrival, and the patient reached the operating room approximately 1 hour and 43 minutes after the emergency call - the earliest verifiable time point, since the exact time of the assault itself was not witnessed (Figure 6). Emergency median sternotomy provided access to all cardiac chambers and enabled rapid hemorrhage control - the standard approach in hemodynamically unstable penetrating cardiac trauma [13,14]. Resuscitative thoracotomy remains an option in selected cases of traumatic cardiac arrest, though its success is contingent on highly trained teams operating within established protocols [17,18].

The relationship between elapsed time and outcome in penetrating cardiac trauma is not strictly linear. Forensic literature shows that even an unequivocally fatal 2 cm left ventricular stab wound can allow tens of seconds of purposeful physical activity before collapse, and that precise prediction of survival time from wound characteristics alone is not possible [19]. In one large autopsy series of fatal penetrating cardiac injuries, mean pre-hospital survival time was considerably longer after stab wounds (approximately 202 minutes) than after gunshot wounds (approximately 30 minutes), reflecting a slower, partially self-limiting rate of haemorrhage with sharp, lower-energy weapons [5]. This physiological window helps explain why, in the present case, an in-hospital delay of roughly 50 minutes between arrival and skin incision-driven by operating theatre availability rather than by any failure of recognition-did not preclude survival: tamponade itself transiently limited further haemorrhage, eFAST-directed triage avoided diagnostic delay, and damage control resuscitation (massive transfusion protocol, tranexamic acid, titrated vasopressor support) maintained coronary and cerebral perfusion while definitive surgical control was awaited. Comparable resilience to delay has been reported elsewhere: a recent case series of penetrating left ventricular injuries described survival after intervals of approximately two hours (stab wound adjacent to the left anterior descending artery) and seven hours (gunshot wound, delayed by a concurrent mass-casualty incident, with intraoperative cardiac arrest successfully managed) (Table 1) [20]. Taken together, these observations support the principle that, once tamponade physiology is established, and airway and circulatory support are maintained, moderate delay to definitive repair can be survived provided resuscitation is continuous and effective-though this should never be read as licence to defer surgical source control, which remains the single most time-critical intervention in this condition [7,10,11].

**Table 1:** Comparative summary of penetrating left ventricular injuries with documented time-to-surgery, operative approach, and outcome AAST: American Association for the Surgery of Trauma; LAD: left anterior descending artery; LV: left ventricle

Source	Mechanism/site	Time: injury to surgery	CPB	Associated findings	Outcome
Present case (2026)	Stab, left precordium; transmural LV laceration (AAST IV)	~1 h 43 min (call to incision)	Not used	Coronary arteries visualised, uninvolved; lung parenchyma intact	Survived; discharged Day 11
Isaza-Restrepo et al., 2023 [5] (autopsy series, n=261)	Mixed stab/gunshot	Mean 202 min (stab) vs 30 min (gunshot), pre-hospital	-	Lung involved in 66% of fatal cases	65% died before reaching medical care
Franchi et al., 2016 [19]	Stab, LV, 2 cm laceration	No medical intervention	-	Forensic case; brief purposeful activity before collapse	Fatal
Pswarayi et al., 2025 (Case 1) [20]	Stab, anterior LV, adjacent to LAD (AAST V)	~2 h	Not reported	LAD injury; coronary-sparing mattress sutures	Survived; discharged Day 13 (postoperative Takotsubo cardiomyopathy)
Pswarayi et al., 2025 (Case 2) [20]	Gunshot, posterior LV (AAST V)	~7 h (mass-casualty delay)	Not reported	Associated liver and diaphragm injury; intraoperative cardiac arrest	Survived after resuscitation; discharged on Day 8

The mechanism of injury also merits comment, since it plausibly explains both the cardiac and the pulmonary findings. The entry wound lay 2 cm medial to the left nipple, in the fourth intercostal space, within the anatomical "cardiac box" and directly overlying the pericardium at a point where the anterior margin of the left lung is normally retracted laterally by the cardiac notch - an anatomical relationship that offers a plausible explanation for why the blade reached the pericardium and left ventricle without lacerating the underlying lung parenchyma, confirmed intact on direct intraoperative inspection. The left hemopneumothorax seen on eFAST is attributable to the open chest wound itself, which allowed air entry into the pleural space, together with blood tracking from the wound tract and pericardial space into the pleural cavity, rather than to a parenchymal lung injury. This is a fortunate rather than an expected finding: associated lung injury is common with wounds in this anatomical region, present in roughly two-thirds of fatal cases in one large autopsy series [5], and its absence here is likely to have preserved pulmonary function at a critical moment, contributing to the favourable outcome.

Operative survival, however, is only the first step. This patient arrived in the ICU with profound metabolic acidosis, obstructive and hemorrhagic shock, and high vasopressor requirements. Damage control resuscitation, encompassing balanced blood product transfusion, invasive hemodynamic monitoring, acidosis correction, and gradual vasoactive withdrawal, was essential to reverse shock physiology and prevent secondary organ injury [21,22].

What is less often discussed in penetrating cardiac trauma is what follows the acute phase. Survivors remain at risk of recurrent bleeding, coagulopathy, myocardial dysfunction, arrhythmias, pseudoaneurysm formation, and multi-organ failure [21,22]. Beyond these, reduced functional capacity, neurocognitive impairment following prolonged shock, and post-traumatic stress disorder represent sequelae that surgical repair alone cannot address [23,24]. Echocardiographic surveillance, cardiac rehabilitation, and psychosocial support are frequently required to optimize recovery [25]. Successful management should therefore be defined not by operative survival alone, but by restoration of meaningful functional status and quality of life.

This case illustrates that survival with favorable outcome following penetrating left ventricular injury is achievable when prehospital recognition, rapid surgical control, postoperative critical care, and long-term multidisciplinary follow-up are effectively integrated within an organized trauma system.

## Conclusions

Penetrating cardiac injuries remain among the most time-critical emergencies in trauma care. Favorable outcomes are closely associated with an integrated sequence of actions: early recognition at the scene, rapid transport, prompt diagnosis, and surgical intervention within centers experienced in thoracic trauma management. This case illustrates the value of coordinated multidisciplinary care involving prehospital services, trauma surgery, and anesthesiology teams, and shows that even injuries with traditionally high mortality - such as penetrating left ventricular lacerations - can result in favorable outcomes through a protocol-driven, coordinated response, even when some delay to definitive surgical control cannot be avoided, provided resuscitation and diagnostic triage are executed promptly and effectively.

This report nonetheless has several limitations that should temper its conclusions. The exact time of injury was not witnessed and was estimated from the emergency call, and the patient could not be reached for structured follow-up after discharge on Day 11, so his longer-term functional, psychological, and echocardiographic status remains unknown. Assessment of recovery from shock relied on a single postoperative blood gas rather than a serial trend, and structures beyond the coronary arteries and left lung - the interventricular septum, cardiac valves, great vessels, and diaphragm - were not specifically inspected or imaged, so subtle injury to them cannot be entirely excluded. As with any single case report, the relative contribution of each element of care to this patient's survival cannot be isolated from the others, and the recommendations above rest on the wider trauma literature cited throughout this report rather than on this case alone. The principal learning point is that, once tamponade physiology is recognised, and diagnostic and resuscitative measures are promptly instituted, survival remains achievable even after a severe, transmural left ventricular injury and a non-trivial delay to definitive surgery. Ongoing training and centralization of severe cardiac trauma management within specialized trauma centers remain essential to optimizing survival in these challenging cases, and future reports should prospectively incorporate structured follow-up so that comparative outcome data can be built rather than reconstructed retrospectively.

## References

[1] Schellenberg M, Inaba K, Bardes JM (2023). Shot through the heart: a 17-year analysis of pre-hospital and hospital deaths from penetrating cardiac injuries. Injury.

[2] Lee C, Jebbia M, Morchi R, Grigorian A, Nahmias J (2025). Cardiac trauma: a review of penetrating and blunt cardiac injuries. Am Surg.

[3] Krammel M, Frimmel N, Hamp T (2024). Outcomes and potential for improvement in the prehospital treatment of penetrating chest injuries in a European metropolitan area: a retrospective analysis of 2009-2017. Injury.

[4] Parentic M, Podolski E, Korda M (2024). Penetrating injury to the left ventricle caused by attempted suicide - a case report. J Surg Case Rep.

[5] Isaza-Restrepo A, Donoso-Samper A, Benitez E (2023). Retrospective analysis of 261 autopsies of penetrating cardiac injuries with emphasis on sociodemographic factors. Sci Rep.

[6] Blatchford JW 3rd (1985). Ludwig Rehn: The First Successful Cardiorrhaphy. Ann Thorac Surg.

[7] Werner OJ, Sohns C, Popov AF, Haskamp J, Schmitto JD (2012). Ludwig Rehn (1849-1930): the German surgeon who performed the worldwide first successful cardiac operation. J Med Biogr.

[8] Duval P (1920). Bradshaw Lecture on the surgery of the heart. Lancet.

[9] Beck CS (1935). Two cardiac compression triads. JAMA.

[10] Mandell SP (2026). Management of cardiac injury in severely injured patients. UpToDate.

[11] Clarke DL, Quazi MA, Reddy K, Thomson SR (2011). Emergency operation for penetrating thoracic trauma in a metropolitan surgical service in South Africa. J Thorac Cardiovasc Surg.

[12] Moore EE, Malangoni MA, Cogbill TH (1994). Organ injury scaling IV: thoracic vascular, lung, cardiac, and diaphragm. J Trauma.

[13] Plummer D, Brunette D, Asinger R, Ruiz E (1992). Emergency department echocardiography improves outcome in penetrating cardiac injury. Ann Emerg Med.

[14] Scalea TM, Rodriguez A, Chiu WC (1999). Focused assessment with sonography for trauma (FAST): results from an international consensus conference. J Trauma.

[15] Maughan BC, Kamat A (2012). Lung carcinoma presenting with pathologic femur fracture and false-positive pregnancy test result. Ann Emerg Med.

[16] Anand T, Hanna K, Kulvatunyou N (2020). Time to tracheostomy impacts overall outcomes in patients with cervical spinal cord injury. J Trauma Acute Care Surg.

[17] Lockey D, Crewdson K, Davies G (2006). Traumatic cardiac arrest: who are the survivors?. Ann Emerg Med.

[18] Davies GE, Lockey DJ (2011). Thirteen survivors of prehospital thoracotomy for penetrating trauma: a prehospital physician-performed resuscitation procedure that can yield good results. J Trauma.

[19] Franchi A, Kolopp M, Coudane H, Martrille L (2016). Precise survival time and physical activity after fatal left ventricle injury from sharp pointed weapon: a case report and a review of the literature. Int J Legal Med.

[20] Pswarayi R, Wineberg D, Kerola A (2025). Penetrating cardiac injuries of the left ventricle - a case series and review of literature. Ann Med Surg (Lond).

[21] Holcomb JB, Tilley BC, Baraniuk S (2015). Transfusion of plasma, platelets, and red blood cells in a 1:1:1 vs a 1:1:2 ratio and mortality in patients with severe trauma: the PROPPR randomized clinical trial. JAMA.

[22] Cannon JW, Khan MA, Raja AS (2017). Damage control resuscitation in patients with severe traumatic hemorrhage: a practice management guideline from the Eastern Association for the Surgery of Trauma. J Trauma Acute Care Surg.

[23] Kong VY, Sartorius B, Clarke DL (2015). The selective conservative management of penetrating thoracic trauma is still appropriate in the current era. Injury.

[24] Soar J, Böttiger BW, Carli P (2021). European Resuscitation Council Guidelines 2021: adult advanced life support. Resuscitation.

[25] Nolan JP, Sandroni C, Cariou A (2025). European Resuscitation Council and European Society of Intensive Care Medicine guidelines 2025 post-resuscitation care. Resuscitation.

